# Adaptation in Gait to Lunar and Martian Gravity Unloading During Long-Term Isolation in the Ground-Based Space Station Model

**DOI:** 10.3389/fnhum.2021.742664

**Published:** 2022-01-12

**Authors:** Alina Saveko, Vitaly Brykov, Vladimir Kitov, Alexey Shpakov, Elena Tomilovskaya

**Affiliations:** ^1^Russian Federation State Scientific Center, Institute of Biomedical Problems of the Russian Academy of Sciences, Moscow, Russia; ^2^Federal Science Center of Physical Culture and Sport (VNIIFK), Moscow, Russia

**Keywords:** motor adaptation, gait, electromyography (EMG), body weight unloading, ground reaction (forces), ground based isolation

## Abstract

The aim of the experiment was to evaluate the adaptive responses of biomechanical and electromyographic parameters to vertical unloading (Lunar—0.15 G and Martian—0.35 G) when walking during the 4-month isolation experiment SIRIUS-19 in the ground-based space station model (GBI). The study involved 6 healthy international crew members of the SIRIUS-19 project aged 34 ± 6.2 years (3 women and 3 men). Body Weight Unloading (BWU) conditions was created by the h/p/cosmos airwalk system. The locomotor test included walking (3.5 ± 0.3 km/h) with a sequential change of BWU modes: 5-min walking with 0% BWU (1 G), 5-min walking with 65% BWU (0.35 G) and 5-min walking with 85% BWU (0.15 G). Ground Reaction Force was recorded by the h/p/cosmos treadmill device. Muscle Lab Model 4000e device was used to record the electromyographic signals of the hip and shin muscles. The locomotor test was performed twice before GBI, monthly during GBI and 1 week after leaving isolation. The results obtained before GBI demonstrate that the changes of support and proprioceptive afferentation signals play significant role in reorganizing of the biomechanical structure of motor acts and the development of new movement patterns. The results of the study are consistent with the previously obtained results of other studies in this direction. Despite the fact that during the GBI the participants of the experiment performed regular physical training, a decrease in the performance indicators values was detected, especially pronounced after 100 days of GBI. This is probably due to limited space of a space station model, as well as the development of a special motor stereotype in it. Noteworthy are the results obtained after the 4th session of the experiment, indicating the effect of sensorimotor learning. We think that the data obtained in this study will be useful in research both in gravitational physiology and in clinical medicine.

## Introduction

Sensory-motor adaptation is the result of the coordinated activity of sensory and motor systems, as well as central integration processes. It is the ability to maintain the accuracy and control of movements, to modify motor commands when the sensory signals change. Any changes in the signals from the leading sensory inputs induce the generation of the corresponding motor commands in the central nervous system, which are transmitted along the descending pathways to different body segments (Shadmehr et al., 2010). In particular, studies in the field of gravitational physiology have demonstrated a significant effect of the level of support and proprioceptive afferentation on the regulation of tonic muscle activity (Kozlovskaya et al., 2007). The withdrawal of the support afferentation leads to the decline of the tonic motor units activity in extensor muscles and the alteration of the motor units recruitment patterns in the spinal cord (Shenkman et al., 2017). Also previously shown that load-related afferent feedback modulates vestibular input and can be used by the central nervous system to modify performance of motions (Fredrickson et al., 1966; Jian et al., 2002; Marsden et al., 2003). This is a key factor in the modification of human locomotor patterns under real (Cavanagh et al., 2010; De Witt and Ploutz-Snyder, 2014; Saveko et al., 2020) and simulated (McCrory et al., 2004, 1997; Sylos-Labini et al., 2013) microgravity conditions. For the first time the concept of influence the plantar cutaneous afferents to modulate the postural-tonic system of mammals evolved from experiments performed in microgravity and dry immersion studies (Grigor’ev et al., 2004; Kozlovskaya et al., 2007). Kozlovskaya et al. (2007) demonstrated that dynamic plantar pressure stimulation during a prolonged absence of weight bearing can attenuate the functional neuromotor degradation associated with chronic unloading (Litvinova et al., 2004). With the use of functional magnetic resonance tomography, plantar pressure stimulation was further shown to activate the primary sensorimotor cortex associated with load-bearing stepping (Kremneva et al., 2013), thus implying a convergence of plantar cutaneous stimuli with the supraspinal control of locomotion.

Currently, studies with a support unloading are especially relevant for space medicine in order to predict movement disorders in partial gravity conditions (Richter et al., 2017). The only experience of locomotion and operator activity on the surface of other planets with different gravity level from the Earth’s gravity level revealed disorders in the movements coordination and functional performance of astronauts: the performance of any motor task was characterized by a significant loss of accuracy and control of movements, an increase in the time of performance of operator tasks, etc. (Scheuring et al., 2008). The reorganizing of the biomechanical structure of motor acts in such conditions, among other things, is caused by the alteration of the ratio between body mass and effort values required for its movement performance, a decrease in the activity of postural muscles, disused earth-specific movements, and the development of new movement patterns with a change in the nature of motor activity in general (Kozlovskaya et al., 1988). To date, there are not enough quantitative data characterizing sensory-motor reactions in conditions with different gravity level from the Earth’s gravity level. At the same time, such knowledge is important in the future interplanetary missions, including to the Moon and Mars. Thus, the study of movement patterns while Lunar (0.1–0.20 G) and Martian (0.30–0.40 G) weight unloading is of particular practical importance (Richter et al., 2017). Moreover, as part of the prospects for interplanetary expeditions, crew members will have to adapt not only to the change in the level of gravity, but also to stay in the specific conditions of the limited space of the spacecraft for a long time, which is accompanied by a change in boundaries of the environment space, which can also effect the manipulation of motor representations (Gangopadhyay et al., 2010; Kim et al., 2018).

We have suggested the hypothesis that the factors of long-term isolation in limited space of a space station model can elicit change in the adaptive responses of biomechanical and electromyographic parameters to partial vertical unloading while walking.

One of the ways to change sensory information related to the support load separately is the Body Weight Unloading (BWU). In this connection, the measurement of biomechanical parameters of natural locomotions (for example, walking) while BWU is used to study the mechanisms of adaptive reactions of the sensory-motor system both in gravitational physiology (Sylos-Labini et al., 2013; Pavei and Minetti, 2016) and in clinical medicine (Apte et al., 2018).

In connection with the above, the purpose of the experiment was to assess the adaptive responses of biomechanical and electromyographic parameters to Lunar (0.15 G) and Martian (0.35 G) vertical unloading while walking during the 4-month isolation in the ground model of the space station.

## Materials and Methods

### Subjects

The study involved 6 healthy crew members (3 women and 3 men) of the international project SIRIUS-19 (Scientific International Research in Unique Terrestrial Station) implemented by IMBP RAS and NASA HRP aged 34 ± 6.2 years (height: 175.0 ± 9.6 cm; weight: 68.8 ± 13.1 kg; body mass index: 22.3 ± 2.9 kg/m2). Inclusion criteria: the crew was to consist of representatives of both sexes, several nationalities and different cultures, lack of experience of space flights. Exclusion criteria: the presence of pathologies of the respiratory, digestive, urinary, sensorimotor, circulatory, nervous and integumentary systems that cause additional risks to health and life during 4 months of isolation in the space station model. The number of participants in the experiment was limited by the planned scenario of a flight to the moon and back of an international crew of six people^1^ (National Aeronautics and Space Administration, 2021). One of the study participants had a previous experience of 3 months of isolation in a mock-up of a Martian research station. Crew members were allowed to participate in the experiment by the medical expert commission of the Institute of Biomedical Problems of RAS and signed an Informed Consent to participate in the study in accordance with the provisions of the Helsinki Declaration of Human Rights. The research procedure was preliminarily reviewed and approved by the Biomedical Ethics Commission of the Institute of Biomedical Problems of RAS. The participants provided their written informed consent to participate in this study.

### Ground-Based Space Station Model

**FIGURE 1:**
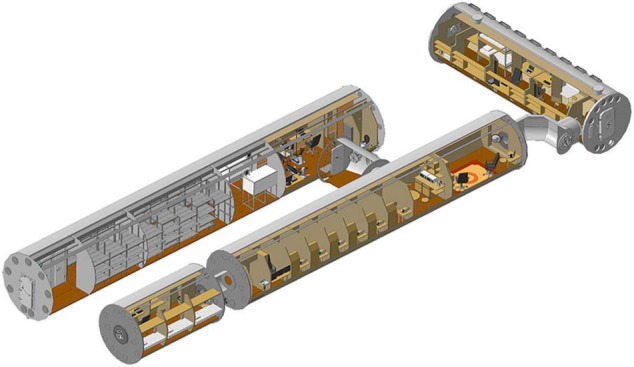
Main view of medical-technical experimental facility. Right side. IBMP/Haider Hobihozin.

The crew members of the SIRIUS-19 project spent 4 months in the medical-technical facility of IBMP RAS what meant for simulation of conditions of life and activity of the crew, that are maximally close to the conditions of real spaceships. Ground-based Isolation (GBI) was accompanied by the control of the experimental parameters (work and rest regime, habitat parameters, nutrition, etc.). It is important to note that during GBI, the crew members performed physical training like on the International Space Station (Kozlovskaya et al., 2015) and were in Earth gravity. The total volume of the modules was 550 m^3^ (Figure 1). The scenario for the 4-month mission SIRIUS-19 included a simulation of a real manned flight to the Moon with a landing on the Lunar surface and return to the Earth. It is worth noting that studies using this model are relevant not only for predicting risks in long-term space flights, but also for patients who are in prolonged isolation and/or conditions of reduced motor activity (Mitrokhin et al., 2020).

### Body Weight Unloading System

**FIGURE 2:**
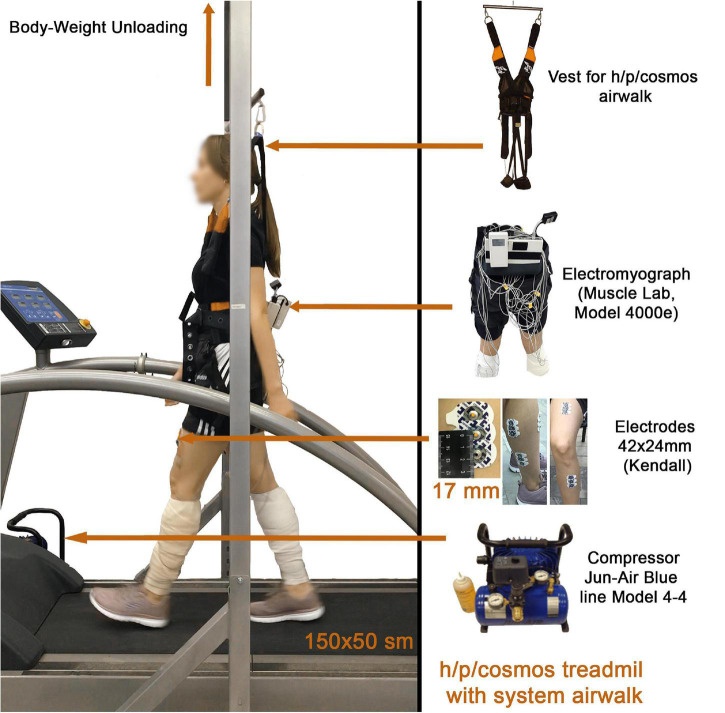
Equipment used during a session of experiment.

Walking was carried out on the treadmill H/P/Cosmos Mercury 4.0 (H/P/Cosmos sports & medical gmbh, Germany) with a canvas size of 150 × 50 cm. The body weight of the subjects was determined before each session of the experiment using Kistler force plate (Kistler Group, Switzerland) installed under the treadmill canvas. The change and regulation of the gravitational load on the musculoskeletal system was carried out by vertical unloading using the H/P/Cosmos-Air Walk system (H/P/Cosmos sports and medical gmbh, Germany), consisting of the Jun-Air Blue line Model 4-4 compressor, a frame structure and a special H/P/Cosmos-Air Walk vest (Figure 2). Locomotor test included different BWU mode (Martian gravity mode—35% of the Earth’s weight and Lunar gravity—15% of the Earth’s weight).

### Procedure

The locomotor test included walking with sequential changes in BWU modes: 5 min walking with 0% BWU (1 G), 5 min walking with 65% BWU (0.35 G) and 5 min walking with 85% BWU (0.15 G). Walking in the active mode of the treadmill was carried out at a constant speed of 3.5 ± 0.3 km/h (0.97 ± 0.08 m/s; 2.17 ± 0.19 mph). This locomotor test was performed 20 and 7 days before GBI (baseline sessions), monthly during GBI and 7 days after GBI. Locomotor test data during GBI were recorded only on the 50th and 100th day of GBI (on the 2nd and 4th months of isolation). The mode change was carried out and controlled by the performer of the experiment (before and after GBI—by the researcher, during GBI—by one of the crew members). There was no rest period between mode changes. All experiment sessions were conducted in the space station model module. Each experimental session was accompanied by a video recording of the performance of the locomotor test in order to control the protocol of the study. Quantitative evaluation of video data was not carried out, but it was used for observational evaluation of movements based on the obtained quantitative data of biomechanical parameters and electromyographic activity of muscles.

### Biomechanical Parameters Measurement

**FIGURE 3:**
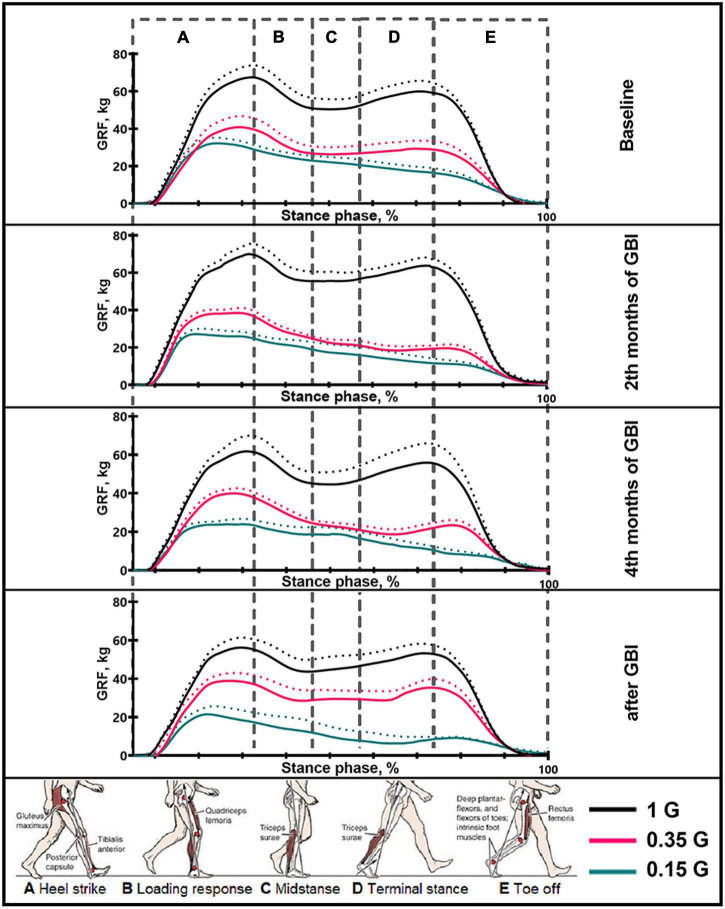
The average podogram of 6 SIRIUS-19 crew members in different sessions of the experiment. On the ordinate axis: ground reaction force, kg. On the abscissa axis: stance phase, %. 1 G, 0.35 G, 0.15 G—body weight unloading modes—0%, 65%, 85% of the Earth weight, respectively. «Baseline»—results obtained before isolation. «2 months of GBI» and «4 months of GBI»—results obtained on the 50th and 100th day of isolation respectively. «1 week after GBI»—results obtained a week after leaving isolation. The dotted lines display the error bars (SEM).

Ground Reaction Force (GRF) were recorded by the Kistler force plate using the “Kistler Gateway” software (Kistler Group, Switzerland). The treadmill was equipped with two force plate under the canvas, arranged in the front and back of area of movement. Each force plate contained four piezoelectric uniaxial load sensors that measure the vertical component of the support reaction (Ivanenko et al., 2002). In our study, data from load sensors were recorded at a frequency of 100 Hz using software provided by the treadmill developer. At the end of the 3rd minute from the beginning of the unloading mode change, biomechanical parameters were recorded 3 times with a duration of 30 s. Based on the data obtained, GRF (in kg) parameters were calculated using the “Kistler Gateway” software at different moments of the stance phase: Heel strike, Loading response, Midstance, Terminal stance, Toe off (Figure 3). The value of the amplitude of Midstance was considered as the difference between the GRF value of the minimum vertical pressure at the moment of Midstance and the average value of the GRF of the maximum vertical pressure at the moments of Heel strike and Toe off. Stance phase is defined as the period of time where the foot is in contact with the ground. Previous studies show that quantitative assessment at different moments of stance (also known as “inner-stance phases”) can bring additional insight into gait assessment (Burnfield, 2010; Mariani et al., 2013; Vaverka et al., 2015). As well as the temporal and spatial characteristics of walking were calculated “Kistler Gateway” software: Gait cycle time (s), Single support time (s), Double support time (s), Step length (cm), Cadence (steps/min). This the temporal characteristics of walking is commonly used in such studies (Awai et al., 2017; Apte et al., 2018).

### Electromyography Measurement

**FIGURE 4:**
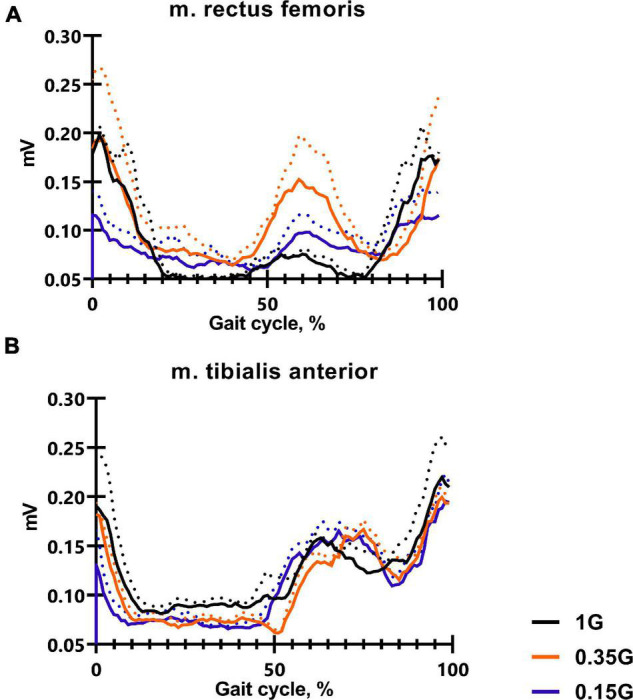
The average EMG activity profile of 6 SIRIUS-19 crew members in baseline sessions: m. rectus femoris **(A)**, m. tibialis anterior **(B)**. On the ordinate axis: EMG amplitude, mV. On the abscissa axis: gait cycle, %. 1 G, 0.35 G, 0.15 G—body weight unloading modes—0%, 65%, 85% of the Earth weight respectively. «Baseline»—results obtained before isolation. «2 months of GBI» and «4 months of GBI»—results obtained on the 50th and 100th day of isolation, respectively. «1 week after GBI»—results obtained a week after leaving isolation. The dotted lines display the error bars (SEM).

The electromyographic activities were recorded by means of disposable surface electrodes bipolar skin electrodes 42 × 24 cm Kendalll, which were located in the middle of the projection of the belly of muscles. The distance between the electrodes was 17 mm. The surface of the skin was carefully cleaned with a sponge, and then degreased with ethanol before applying the electrodes, in order to reduce the electrical resistance. EMGs were recorded using a MuscleLab 4000e telemetric eight-channel hardware—software complex, which allows the EMG-activity of muscles to be recorded at a distance of 100 m. Based on the Bluetooth wireless technology, the data were recorded online on a computer hard drive. The input impedance of each channel of an electromyograph was 2 MΩ at a transmission frequency of an EMG signal of 100 Hz on one Bluetooth channel. The MuscleLab 4000e hardware allows inverting and averaging the electromyographic signal during a 10-ms exposure. During processing the inverted EMG was smoothed using a Butterworth low-pass filter of the second order. The electromyographical signals of the leg muscles were integrated, and the mean and standard deviations were calculated from 13–15 double steps (Figure 2). Electromyographic activity was recorded from 4 muscles of the leg and thigh: m. soleus (SL), m. gastrocnemius lateralis (GL), m. tibialis anterior (TA) and m. rectus femoris (RF). Based on the results of similar studies, muscle activity was considered in terms of the magnitude of the EMG signal as an average value over the entire gait cycle. Also the profiles of EMG activity of each muscles during the gait cycle were analyzed (Awai et al., 2017; Apte et al., 2018; Figures 4, 7).

**FIGURE 5:**
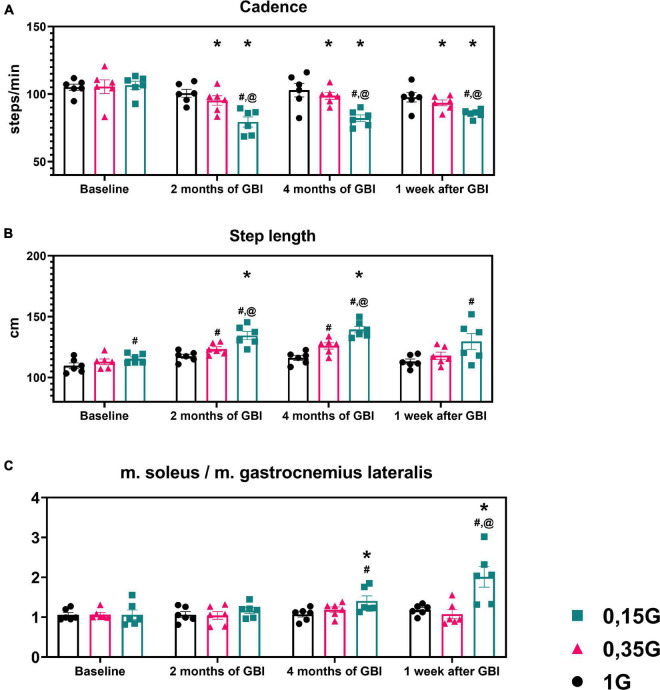
Kinematic characteristics of walking in different sessions of the experiment: Step length **(A)** in centimeters, Cadence **(B)** in steps per minute. **(C)** The ratio of the average amplitude of EMG activity of m. soleus to the average amplitude of EMG activity of m. gastrocnemius lateralis. 1 G, 0.35 G, 0.15 G—body weight unloading modes—0%, 65%, 85% of the Earth weight respectively. «Baseline»—results obtained before isolation. «2 months of GBI» and «4 months of GBI»—results obtained on the 50th and 100th day of isolation, respectively. «1 week after GBI»—results obtained a week after leaving isolation. *A significant difference compared to the baseline values. ^#^A significant difference compared with values at 1 G. ^@^A significant difference compared with values at 0.35 G. ${}^{N\,\underline{o}}$A significant difference compared with values at 0.15 G. Data presented as MEAN with SEM + individual results.

**FIGURE 6:**
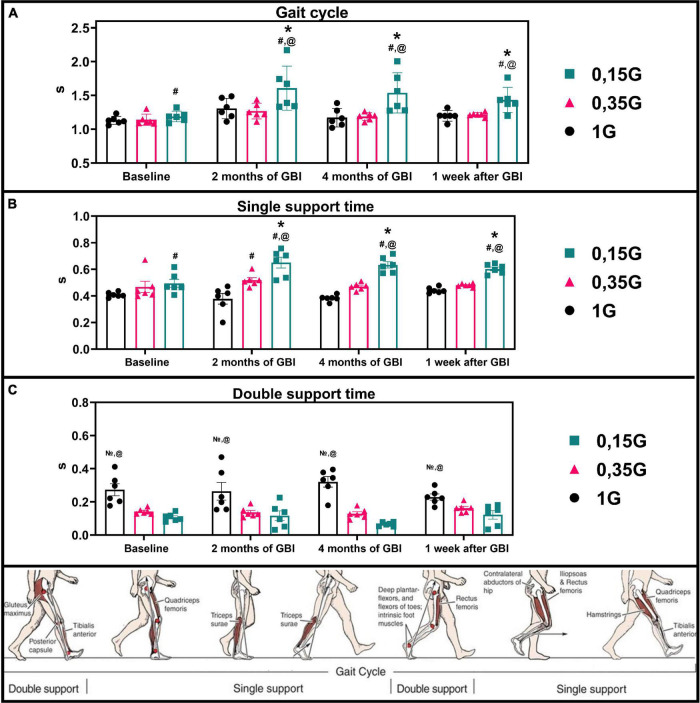
Kinematic characteristics of walking in different sessions of the experiment: Gait cycle **(A)**, Single support **(B)**, Double support **(C)** in seconds. 1 G, 0.35 G, 0.15 G—body weight unloading modes—0%, 65%, 85% of the Earth weight, respectively. «Baseline»—results obtained before isolation. «2 months of GBI» and «4 months of GBI»—results obtained on the 50th and 100th day of isolation respectively. «1 week after GBI»—results obtained a week after leaving isolation. *—a significant difference compared to the baseline values. ^#^—a significant difference compared with values at 1 G. ^@^—a significant difference compared with values at 0.35 G. ${}^{N\,\underline{o}}$—a significant difference compared with values at 0.15 G. Data presented as MEAN with SEM + individual results.

**FIGURE 7:**
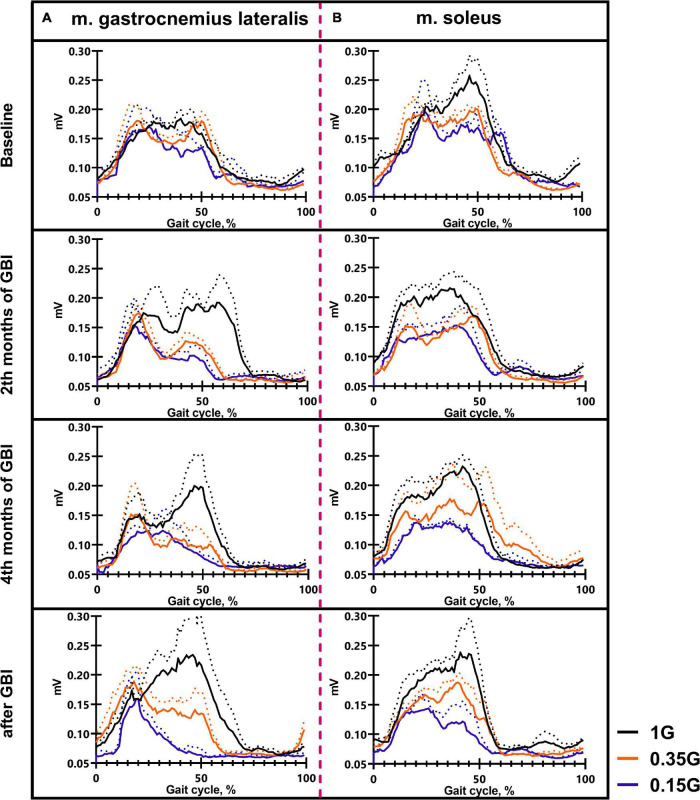
The average EMG activity profile of 6 SIRIUS-19 crew members in different sessions of the experiment: m. gastrocnemius lateralis **(A)**, m. soleus **(B)**. On the ordinate axis: EMG amplitude, mV. On the abscissa axis: gait cycle, %. 1 G, 0.35 G, 0.15 G—body weight unloading modes—0%, 65%, 85% of the Earth weight, respectively. «Baseline»—results obtained before isolation. «2 months of GBI» and «4 months of GBI»—results obtained on the 50th and 100th day of isolation, respectively. «1 week after GBI»—results obtained a week after leaving isolation. The dotted lines display the error bars (SEM).

### Statistical Analysis

When analyzing the results of the study, the difference of parmaterials in both between the experiment sessions (before GBI, on the 2nd and 4th month of GBI, after GBI) and between different BWU modes (1 G, 0.35 G, 0.15 G) was estimated. For data analysis, we used the GraphPad Prism version 8 (GraphPad Software, United States). The data were analyzed using Two Way Repeated Measures ANOVA, *post hoc* Bonferroni. All significance levels were set at *p* ≤ 0.05. All data were tested for normality using Shapiro–Wilk test (all data are normally distributed—*p* > 0.05). The results of two baseline (pre-GBI) experimental sessions were averaged and had no statistical difference between them. The values that have reached a significant difference compared with the baseline values in the text are marked with a “*” sign, compared with the values at 0% BWU (1 G)—“^#^,” compared with the values at 65% BWU (0.35 G)—“^@^,” compared with values at 85% BWU (0.15 G)—“${}^{N\,\underline{o}}$.”

## Results

### 0% Body Weight Unloading (1 G)

The results obtained in the baseline studies were completely consistent with normal walking in both EMG profile of muscle activity and biomechanical characteristics (Latash and Zatsiorsky, 2015). All the moments of the step were well expressed on the walking podogram (Figure 3). At the same time, GRF values at the moment of Heel strike were higher than GRF values at the moment of Toe off by 7.49 ± 5.42 kg, and Midstance amplitude was 13.29 ± 4.86 kg (Figure 3). Gait cycle time was 1.13 ± 0.08 s, Cadence was 105.05 ± 3.54 steps/min, Step length was 109.69 ± 2.11 cm, Double support time was 0.27 ± 0.03 s, and Single support time was 0.40 ± 0.11 s (Figures 5A,B, 6). Equal work of the flexor and extensor muscles of the lower extremities was also observed (Figures 4, 7).

During GBI, there was a tendency toward an increase in stride length, a decrease in stride frequency (Figures 5A,B). At the same time, the difference between GRF values at Heel strike moment and Toe off moment decreased: on the 2nd month this difference was 6.46 ± 4.16 kg, and on the 4th month—2.65 ± 1.05* kg (*p* = 0.0384; *F* = 7.843; Cohen’s *d* = 2.523). It is interesting to note that on the 2nd month of GBI, the value of the amplitude of Midstance decreased to 10.91 ± 3.53 kg against the background of a tendency toward a decrease in EMG activity of SL and an increase in Gait cycle time. Also on the 4th month the value of the amplitude of Midstance increased to 17.57 ± 3.12* kg (*p* = 0.0015; *F* = 39.23; Cohen‘s *d* = 5.615) against the background of a tendency toward increased EMG activity of SL (Figures 3, 6, 7).

After GBI, EMG activity of SL and GL muscles increased (Figure 7), while the overall GRF values decreased by 6.54 ± 2.17* kg (*p* = 0.0071; *F* = 19.32; Cohen‘s *d* = 3.013). In particular, GRF values at Heel strike moment was 11.37 ± 2.23* kg (*p* = 0.0262; *F* = 9.854; Cohen‘s *d* = 5.098) lower and GRF values at Toe off moment—was 6.52 ± 1.87* kg (*p* = 0.047; *F* = 6.824; Cohen‘s *d* = 3.486) lower compared to pre-GBI data. The difference between the GRF values at these moments was 3.01 ± 2.01* kg (*p* = 0.0474; *F* = 6.867; Cohen‘s *d* = 1.497), and the value of the amplitude of Midstance was 10.34 ± 3.91 kg. It is worth noting that during the experiment, the moment of the minimum vertical pressure at Midstance gradually shifted closer to the beginning of the gait cycle. This phenomenon is most pronounced on the 4th month of GBI and on the 7th day after it. Also on the 7th day after GBI, the moment of maximum vertical pressure at Heel Strike occurred faster than in the baseline studies (Figure 3).

### 65% Body Weight Unloading (0.35 G)

In the background sessions, the most notable changes in comparison with 1 G were recorded in the total GRF values and EMG activity of the extensor muscles—SL and GL. The total GRF values at 0.35 G were lower than the total GRF values at 1 G by 22.10 ± 3.44^#^ kg/45.29 ± 6.32^#^% (*p* = 0.0003; *F* = 81.15; Cohen‘s *d* = 6.424). At the same time the difference between GRF values at Heel strike moment and Toe off moment was higher compared to 1 G and amounted to 11.56 ± 4.76 kg, also the value of the amplitude of Midstance was lower and amounted to 8.22 ± 2.13^#^ kg (*p* = 0.0050; *F* = 22.78; Cohen‘s *d* = 3.859). All the moments of gait were well expressed on the walking podogram, as at 1 G, however, the moment of maximum vertical pressure at Heel Strike came earlier (Figure 3). Gait cycle time was 1.13 ± 0.03 s, Cadence was 105.45 ± 11.01 strides/min, Step length was 112.98 ± 7.23 cm, Double support time was 0.14 ± 0.01^#^ s (*p* = 0.0174; *F* = 12.22; Cohen‘s *d* = 5.813), and Single support time was 0.47 ± 0.02 s (Figures 5A,B, 6).

During GBI, a significant decrease in GRF values at Heel Strike moment wasn‘t revealed, however, GRF values at Toe Off moment were lower than in the baseline sessions at 0.35 G by 9.5 ± 1.76^#^ kg (*p* = 0.0269; *F* = 6.902; Cohen‘s *d* = 5.397), 5.98 ± 2.04^#^ kg (*p* = 0.0217; *F* = 7.653; Cohen‘s *d* = 2.931) and 1.08 ± 0.94^#^ kg (*p* = 0.0383; *F* = 5.790; Cohen‘s *d* = 1.148) on the 2nd, 4th month of GBI and after it, respectively. At the same time, the difference between GRF values at Heel strike moment and Toe off moment gradually decreased, and on the 7th day after GBI difference was 11.04 ± 6.03 kg. Midstance amplitude was practically unnoticeable on podogram in the 2nd and 4th months of GBI and was noticeably shifted toward the end of the gait cycle. On the 7th day after GBI Midstance amplitude was 10.01 ± 3.11 kg (Figure 3).

The dynamics of kinematic and electromyographic characteristics during GBI at 0.35 G was similar to the dynamics of characteristics at 1 G, but was more pronounced. So for example, on the 2nd month GBI Step length increased by 10.50 ± 3.67^#^ cm (*p* = 0.0337; *F* = 8.425; Cohen‘s *d* = 2.861), Cadence decreased by 10.23 ± 2.14* steps/min (*p* = 0.0164; *F* = 12.59; Cohen‘s *d* = 4.780), and Single support time significantly increase by 0.05 ± 0.01* s (*p* = 0.0149; *F* = 13.26; Cohen‘s *d* = 5.364) compared to pre-GBI results (Figures 5A,B, 6).

The dynamics of EMG activity of the muscles at 0.35 G is of particular interest. In the baseline sessions EMG activity of RF increased by 5.61 ± 8.13^#^% (*p* = 0.0173; *F* = 11.82; Cohen‘s *d* = 0.690), while EMG activity of the extensor muscles SL and GL decreased by 6.57 ± 4.23% and 14.4 ± 3.41^#^% (*p* = 0.0395; *F* = 7.657; Cohen‘s *d* = 4.223), respectively. During GBI and after it, EMG activity of the flexor muscles RF and TA remained practically unchanged; however, EMG activity of the extensor muscles SL and GL sharply decreased. On the 4th months of GBI, EMG activity of SL decreased by 31.09 ± 9.12^*#^% (*p = 0.0019; *F* = 11.61; Cohen‘s *d* = 3.409 and ^#^*p* = 0.0141; *F* = 13.61; Cohen‘s *d* = 1.369), and EMG activity of GL decreased by 22.87 ± 5.89^*#^% (*p = 0.0396; *F* = 7.651; Cohen‘s *d* = 3.882 and ^#^*p* = 0.0010; *F* = 47.87; Cohen‘s *d* = 4.276) compared to 1 G values. After GBI, there was a tendency to an increase in EMG activity of the extensor muscles like at 1 G (Figures 4, 7).

### 85% Body Weight Unloading (0.15 G)

At 0.15 G, the mentioned BWU effects was more pronounced. In baseline sessions the total GRF values sharp decreased by 28.52 ± 2.42^#^ kg/58.44 ± 4.11^#^% (*p* < 0,0001; *F* = 159.4; Cohen‘s *d* = 11.785) compared to 1 G results and by 6.42 ± 1.86^@^ kg/13.15 ± 2.75^@^% (*p* = 0.0039; *F* = 25.61; Cohen‘s *d* = 3.451) compared to 0.35 G results. The podogram was noticeable only at Heel Strike moment, that shifted to the beginning of the gait cycle. Toe off and Midstance were not expressed on the podogram (Figure 3). Gait cycle time was 1.19 ± 0.21^#^ s (*p* = 0.0355; *F* = 8.169; Cohen‘s *d* = 0.377), Cadence was 106.51 ± 8.56 steps/min, Step length was 116.55 ± 9.03^#^ cm (*p* = 0.0116; *F* = 15.07; Cohen‘s *d* = 1.046), Double support time was 0.10 ± 0.03^#^ s (*p* = 0.0067; *F* = 19.87; Cohen‘s *d* = 5.666), and Single support time was 0.49 ± 0.02^#^ s (*p* = 0.0413; *F* = 7.448; Cohen‘s *d* = 1.138) (Figure 6).

During GBI, the total GRF values gradually decreased in comparison with the baseline values, and on the 7th day after GBI, the total GRF values were lower than the baseline ones by 9.09 ± 2.08* kg (*p* = 0.0061; *F* = 20.65; Cohen‘s *d* = 4.370) (Figure 3). At the same time, during GBI, the kinematic characteristics of walking significantly changed compared to the baseline values. On the 2nd month of GBI, Step length sharply increased by 18.68 ± 8.84^*#@^ cm (*p = 0.0004; *F* = 70.74; Cohen‘s *d* = 2.113; ^#^*p* = 0.0077; *F* = 18.50; Cohen‘s *d* = 4.301; ^@^*p* = 0.0154; *F* = 13.01; Cohen‘s *d* = 3.605), this parameter continued to increase, and on the 4th month of GBI, it was 22.87 ± 6.73^*#@^ cm (*p = 0.0005; *F* = 64.98; Cohen‘s *d* = 3.398; ^#^*p* = 0.0185; *F* = 11.80; Cohen‘s *d* = 1.125; ^@^*p* = 0.0093; *F* = 16.82; Cohen‘s *d* = 0.410) higher than before the isolation. Cadence values had the reverse tendency to Step length values and decreased on the 2nd month GBI—by 27.14 ± 9.48^*#@^ steps/min (*p = 0.0005; *F* = 64.98; Cohen‘s *d* = 2.862; ^#^*p* = 0.0185; *F* = 11.80; Cohen‘s *d* = 1.125; ^@^*p* = 0.0093; *F* = 16.82; Cohen‘s *d* = 0.410), on the 4th month GBI—by 24.35 ± 7.11^*#@^ steps/min (*p = 0.0001; *F* = 111.7; Cohen‘s *d* = 3.424; ^#^*p* = 0.0119; *F* = 14.92; Cohen‘s *d* = 1.339; ^@^*p* = 0.0010; *F* = 46.74; Cohen‘s *d* = 0.683). In addition, Gait cycle time increased mainly due to an increase in Single support time. Gait cycle time increased on the 2nd month GBI—by 0.42 ± 0.12^*#@^ s (*p = 0.0241; *F* = 10.22; Cohen‘s *d* = 3.513; ^#^*p* = 0.0478; *F* = 5.39; Cohen‘s *d* = 0.231; ^@^*p* = 0.0225; *F* = 10.62; Cohen‘s *d* = 0.325), on the 4th month GBI—by 0.35 ± 0.07^*#@^ s (*p = 0.0487; *F* = 6.72; Cohen‘s *d* = 5.258; ^#^*p* = 0.0407; *F* = 7.513; Cohen‘s *d* = 0.273; ^@^*p* = 0.0212; *F* = 10.98; Cohen‘s *d* = 0.331). On the 7th day after the isolation, these parameters did not return to pre-GBI values (Figures 5A,B, 6).

In all experimental sessions at 0.15 G, EMG activity of the extensor muscles SL and GL was significantly lower compared to 1 G, and the moment of the maximum EMG activity of these muscles was noticeably shifted toward the beginning of the gait cycle. In baseline sessions EMG activity of the extensor muscles SL and GL was 16.84 ± 7.33^#^% (*p* = 0.0132; *F* = 14.37; Cohen’s *d* = 2.297) and 12.79 ± 8.12^#^% (*p* = 0.0216; *F* = 8.13; Cohen’s *d* = 1.587) lower compared to 1 G results and 2.74 ± 2.87% and 6.67 ± 5.67% compared to 0.35 G results, respectively (Figure 7). During GBI, this difference increased, and on the 4th month of GBI, EMG activity of SL and GL was lower by 27.24 ± 4.42^*#^% (*p = 0.0001; *F* = 87.11; Cohen’s *d* = 6.162; and ^#^*p* = 0.00126; *F* = 13.43; Cohen’s *d* = 2.143) and 24.19 ± 3.76^*#^% (*p = 0.0013; *F* = 56.67; Cohen’s *d* = 6.433; and ^#^*p* = 0.0234; *F* = 7.852; Cohen’s *d* = 1.562) compared to 1 G results, respectively. After GBI EMG activity of SL and GL continued to increase and was lower by 31.17 ± 3.13^*#^% (*p = 0.0034; *F* = 27.01; Cohen’s *d* = 9.958; and ^#^*p* = 0.0381; *F* = 6.837; Cohen’s *d* = 1.133) and 42.17 ± 6.17^*#^% (*p < 0,0001; *F* = 142.8; Cohen’s *d* = 6.834; ^#^*p* = 0.0311; and *F* = 8.513; Cohen’s *d* = 1.614) compared to 1 G results and by 16.65 ± 6.73^*@^% (*p = 0.0212; *F* = 7.866; Cohen’s *d* = 2.473; and ^@^*p* = 0.0411; *F* = 5.372; Cohen’s *d* = 1.161) and 28.81 ± 6.13^*@^% (*p = 0.0121; *F* = 14.11; Cohen’s *d* = 4.699; ^@^*p* = 0.0267 and *F* = 8.145; Cohen’s *d* = 1.775) compared to 0.35 G results, respectively (Figures 4, 7).

Of interest is the dynamics of the ratio of the values of EMG activity of SL and GL: in baseline studies, this indicator was 1.06 ± 0.29, on the 2nd month GBI—1.16 ± 0.19, on the 4th month GBI—1.42 ± 0.85^*#^ (*p = 0.0002; *F* = 101.5; Cohen’s *d* = 10.117; ^#^*p* = 0.00181 and *F* = 11.97; Cohen’s *d* = 3.463), on the 7 days after GBI—2.18 ± 0.89^*#@^ (*p = 0.0059; *F* = 21.07; Cohen’s *d* = 4.589; ^#^*p* = 0.00177; *F* = 12.11; Cohen’s *d* = 3.489; ^@^*p* = 0.0070; *F* = 19.39; Cohen’s *d* = 4.419) (Figure 5C).

## Discussion and Conclusion

### Pre-ground-Based Isolation Data

**FIGURE 8:**
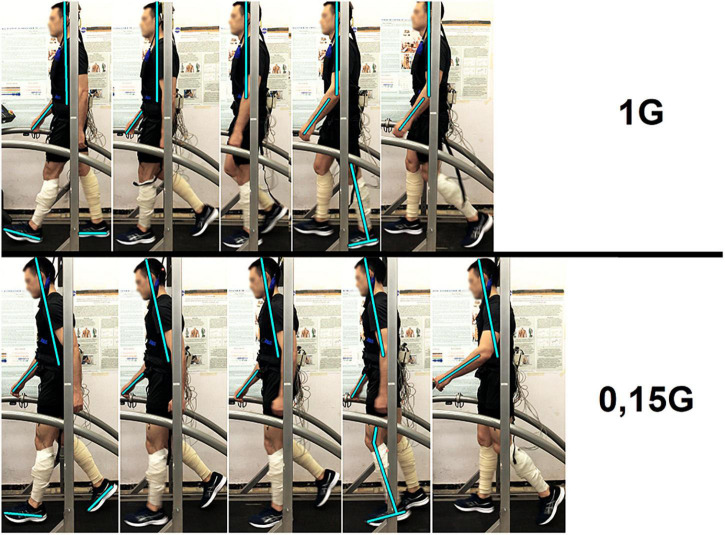
Participant of the experiment at different moments of the step: Heel strike, Loading response, Midstance, Terminal stance, Toe off. 1 G, 0.15 G—body weight unloading modes—0%, 85% of the Earth weight, respectively.

The results obtained before GBI while walking in Martian and Lunar BWU modes (0.35 G and 0.15 G) characterize motor responses during G-transitions. Figure 8 clearly demonstrates the features of the motor strategy when walking in BWU Lunar mode (0.15 G): a tilt of the trunk forward over the support surface, an increased amplitude of the arms swing, an earlier Toe off moment and flexion in the ankle joint at Terminal stance moment. These observations are consistent with changes in the studied parameters while BWU: for example, the shift of the moment of the maximum vertical pressure at Heel Strike to the beginning of the gait cycle, the decrease in EMG activity of the extensor muscles. Despite the significantly decreased repulsion force from the support surface at the Toe off moment, the swing phase increased on the background of a decrease in both single-support and double-support phases due to an increase in the step length and a decrease in the frequency of steps. Also, an increase in the duration of the swing phase is consistent with an increase in the EMG activity of the muscles involved in this phase: mm. tibialis anterior and rectus femoris. Probably, the primary triggering mechanism of these changes is the modification of the signals of the support and proprioceptive afferent, which in turn is a trigger for a decrease in the muscle-tonic activity of the extensor muscles (Kozlovskaya et al., 2007), which leads to the locomotion strategy changes. Interesting that similar strategy was observed during space flight. Thus, the tilt of the body increase over the support surface contributes to an increase in the angular moment speed of falling, and facilitates the work of the muscles (Saveko et al., 2020). Decrease of Cadence also reduces energy expenditure (Zarrugh et al., 1974). Pavei G. and Minetti A.E. have shown a decrease in total external work and range of motion of ankle, hip and knee during walking at a speed of 0.86 m/s and at Lunar level of gravitational unloading using BWU (Pavei and Minetti, 2016). The results of the study are consistent with the previously obtained results of other studies in this direction and complement them (Sylos-Labini et al., 2013; Awai et al., 2017; Richter et al., 2017).

### Data Obtained During and After Ground-Based Isolation

The present study involved several limitations. Our results are limited to the small sample sizes inherent to such research and also the absence of the control group. The results obtained confirm the hypothesis, however, the revealed changes in the adaptive reactions of biomechanical and electromyographic parameters to partial vertical unloading when walking during and after GBI are difficult due to these limitations.

Despite the fact that during GBI, the crew members performed physical training as at the International Space Station, after GBI, a decrease in GRF values was observed. Probably, after GBI, the tendency to an increase in EMG activity of the extensor muscles against the background of a decrease in GRF values is result of recruitment of additional motor units, increased firing rates, and/or synchronization (Farina et al., 2004). It is noteworthy that the ratio of the average amplitude of EMG activity of m. soleus to the average amplitude of EMG activity of m. gastrocnemius lateralis increased significantly during isolation. Since the recruiting order of motor units and the sensory inputs activity interrelated (Shigueva et al., 2015)., we assume that these phenomena may be associated with the presence of crew members in a limited space of a space station model, as well as the formation of a special stereotype of movements in this conditions, which is more likely than the influence of hypokinesia in this case. A decrease in GRF values, in turn, was also observed in real space flight conditions associated with muscle disuse (Saveko et al., 2020). In this case, account should be taken of the fact that a decrease in GRF values can also be the result of motor learning (Zimmermann and Bakker, 2019).

Noteworthy are the results obtained after the 4th session of the experiment—after 50 days of GBI. Sharp significant increase in the gait cycle time, a single support time and a step length with significant decrease in the cadence (frequency of steps) are probably associated with the ability to neuroplasticity and sensory-motor adaptation. It has been shown that people trained to adapt to various sensorimotor disturbances can adapt more quickly to a new sensory environment (Mulavara et al., 2009). Based on this observation, a program of sensorimotor training of astronauts has been developed over the past few years, which also includes BWU method. «Sensorimotor training has been shown to improve locomotor adaptability: increasing stability, lowering cognitive cost and reducing the metabolic expenditure during adaptation to discordant sensory conditions» (Bloomberg et al., 2015). It can be said that during the study participants were trained in motor reactions under the conditions of changed sensory environment. We think that the data obtained in this study will be useful in research both in gravitational physiology in preparation for interplanetary missions and in clinical medicine in the development and evaluation of rehabilitation programs.

## Data Availability Statement

The raw data supporting the conclusions of this article will be made available by the authors, without undue reservation.

## Ethics Statement

The studies involving human participants were reviewed and approved by Biomedical Ethics Commission of the Institute of Biomedical Problems of RAS. The patients/participants provided their written informed consent to participate in this study. Written informed consent was obtained from the individual(s) for the publication of any potentially identifiable images or data included in this article.

## Author Contributions

ASa, VB, VK, and ET designed and conducted the research. ASh helped to process the electromyography data. ASa analyzed the data and drafted the manuscript. ET contributed in the global revision of the manuscript and was a supervisor of the experiment. ASa had primary responsibility for the final content. All authors interpreted the data and have read and approved the final submitted manuscript.

## Conflict of Interest

The authors declare that the research was conducted in the absence of any commercial or financial relationships that could be construed as a potential conflict of interest.

## Publisher’s Note

All claims expressed in this article are solely those of the authors and do not necessarily represent those of their affiliated organizations, or those of the publisher, the editors and the reviewers. Any product that may be evaluated in this article, or claim that may be made by its manufacturer, is not guaranteed or endorsed by the publisher.

## Footnotes

1
https://www.nasa.gov/content/sirius
